# Identifications of Surfactin-Type Biosurfactants Produced by *Bacillus* Species Isolated from Rhizosphere of Vegetables

**DOI:** 10.3390/molecules28031172

**Published:** 2023-01-25

**Authors:** Attila Bartal, Thu Huynh, Anita Kecskeméti, Mónika Vörös, Orsolya Kedves, Henrietta Allaga, Mónika Varga, László Kredics, Csaba Vágvölgyi, András Szekeres

**Affiliations:** 1 Department of Microbiology, Faculty of Science and Informatics, University of Szeged, Közép Fasor 52, H-6726 Szeged, Hungary; 2 Department of Biotechnology, Faculty of Chemical Engineering, Ho Chi Minh University of Technology (HCMUT), 268 Ly Thuong Kiet Street, District 10, Ho Chi Minh City 72607, Vietnam; 3 Vietnam National University Ho Chi Minh City, Linh Trung Ward, Thu Duc District, Ho Chi Minh City 71351, Vietnam

**Keywords:** surfactin, *Bacillus*, biosurfactant, HPLC-HESI-MS/MS

## Abstract

Surfactins are cyclic lipopeptides consisting of a β-hydroxy fatty acid of variable chain length and a peptide ring of seven amino acids linked together by a lactone bridge, forming the cyclic structure of the peptide chain. These compounds are produced mainly by *Bacillus* species and are well regarded for their antibacterial, antifungal, and antiviral activities. For their surfactin production profiling, several *Bacillus* strains isolated from vegetable rhizospheres were identified by their fatty acid methyl ester profiles and were tested against phytopathogen bacteria and fungi. The isolates showed significant inhibition against of *E. amylovora*, *X. campestris*, *B. cinerea*, and *F. culmorum* and caused moderate effects on *P. syringae*, *E. carotovora*, *A. tumefaciens*, *F. graminearum*, *F. solani*, and *C. gloeosporioides*. Then, an HPLC-HESI-MS/MS method was applied to simultaneously carry out the quantitative and in-depth qualitative characterisations on the extracted ferment broths. More than half of the examined *Bacillus* strains produced surfactin, and the MS/MS spectra analyses of their sodiated precursor ions revealed a total of 29 surfactin variants and homologues, some of them with an extremely large number of peaks with different retention times, suggesting a large number of variations in the branching of their fatty acid chains.

## 1. Introduction

Surfactins are cyclic lipopeptide-type biosurfactants first described in 1968 by Arima et al. [1] and are mainly produced by gram-positive *Bacillus* species, such as *B. subtilis*, *B. pumilus*, *B. mojavensis*, *B. licheniformis*, and *B. amyloliquefaciens* [2,3,4,5,6]. These molecules were isolated in the form of white, needle-shaped crystals and were named due to their potent surface activity properties. Surfactin consists of a hydrophobic β-hydroxy fatty acid chain of variable length (C12–C18) linked to a ring of seven amino acids. The cyclic structure is formed by a lactone bridge connecting the β-hydroxyl functional group of the fatty acid to the C-terminal of the heptapeptide [7]. These compounds show numerous different biological activities, such as anti-mycoplasmic [8], anti-tumour [9], anti-inflammatory [10], and antiviral activities [11]. Owing to their surface effect, the research of surfactins in therapeutical, environmental and agricultural applications is also a subject of increasing interest [12,13,14,15].

Surfactins possess a high variability in their fatty acid chain lengths and amino acid sequences; therefore, they bear numerous variants and isoforms. On the basis of their heptapeptide sequences, 10 naturally produced variants were outlined in a summary written by Bonmatin et al. [7]. More recent studies revealed additional groups of surfactin molecules containing Val in the second amino acid position [16], or esterified forms of Glu (glutamic acid 5-methyl ester—GME) [17] and Asp (aspartic acid 4-methyl ester—AME) [17,18,19,20].

Although a large proportion of this lipopeptide group resulting from its potential variability has been well described, and several characteristic structural variations have been reported, the identified surfactin molecule profiles of the different lipopeptide-producing microorganisms are lacking proper comparison. This could be the first step in building a library of the different bacteria with their respective production profiles, which could be used for targeted cultivation with the intention of selectively promoting the production of one surfactin molecule or group in order to compare their biological effects or to characterise their exact structures by the respective techniques. This article aims to be the start of such aspirations.

Different *Bacillus* species and strains produce distinct variants of surfactins in variable ratios, thus affecting the biological and environmental characteristics of the varying ferment broths. These dissimilarities have been reported, for example, in the case of *B. subtilis*, *B. pumilus*, and *B. licheniformis* [21,22]. The alteration of cultivation parameters, including the application of different carbon sources and metal ions [23] or the pH control of the ferment broth [24], is also proven to influence the total surfactin production of the microorganisms and the proportion of the different variants in their ferment broths. The isolation location of the biosurfactant-producing bacteria may also have an important role on the composition of lipopeptide molecules, which may be a key factor from an agricultural standpoint.

In the present study, a fast and easily evaluable HPLC-HESI-MS method was applied in SIM/SRM mode for simultaneously acquiring both quantitative and qualitative information on the surfactin produced by 25 different *Bacillus* strains isolated from vegetable rhizospheres. The surfactin profilings were performed in depth as much as possible, whereby differentiating 158 congeners from the ferment broth. The isolates were identified at the species level, and their in vitro bactericidal and fungicidal properties were determined against phytopathogen microorganisms.

## 2. Results

### 2.1. Identification of the Isolated Strains

Altogether, 25 strains were isolated from the rhizospheres of five types of vegetables, including tomato, pepper, paprika, carrot, and sweet potato on Hungarian and Serbian agricultural areas. The taxonomic identification was carried out by the Sherlock CAS method based on cellular FAME profiling analysis. This system was already successfully used for the discrimination of closely related *Bacillus* species [25] by applying a Similarity Index (SI) together with the constructed libraries in order to identify the isolates. The SI is an interrelation between analysing FA profiles and the mean FA composition of the library’s database as its match. As a consequence, the isolates were identified as *B. atrophaeus*, *B. cereus*, *B. megaterium*, *B. pumilus*, *B. subtilis*, and *B. velezensis* (Table 1) with a high SI (SI > 0.5) and proper SI separations (>0.1), confirming that these strains are typical isolates with high confidence.

In addition, the primary fatty acid methyl ester compositions in *Bacillus* species are shown in Table 2. Interestingly, FA compositions in *B. cereus*, *B. megaterium*, and *B. pumilus* have been divided into two distinguishable groups, named GC subgroup A and B. These species possess a higher content of branched-odd FAs, including 13:0 iso, 15:0 iso, 15:0 anteiso, 17:0 iso, and 17:0 anteiso, as common features of *Bacillus*’ taxonomy [26]. In our cases, the *B. cereus* and *B. megaterium* isolates belonged to the GC subgroup A, while the *B. pumilus* strains were members of the GC subgroup B within the species.

### 2.2. Quantitative Results of the Total Surfactin Production

**Figure 1:**
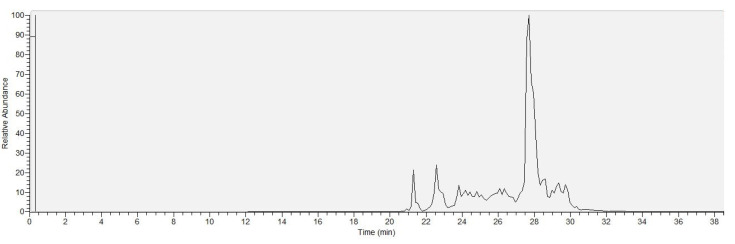
The TIC of the SIM mode measurement of *B. velezensis* strain SZMC 24980.

The lipopeptide concentration in the ferment broth of each sample was carried out after calibration with surfactin standard by calculating the integrated peak areas of the total ion chromatograms (TIC) measured in SIM mode set to the *m*/*z* values of the sodiated precursor ions (Figure 1).

**Figure 2:**
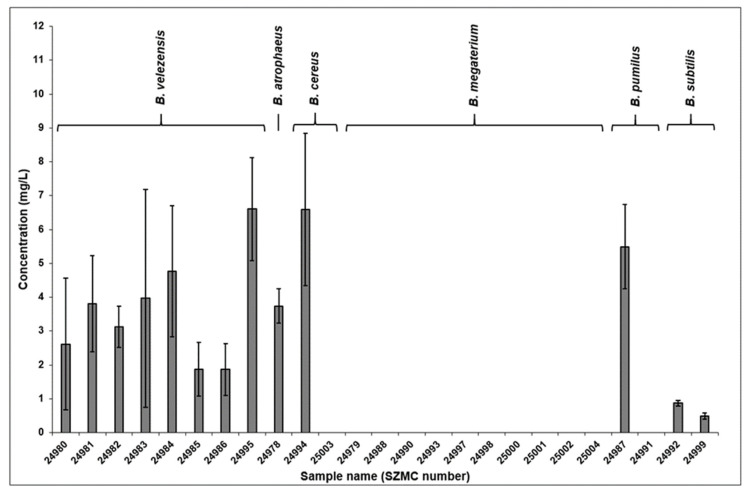
Concentrations of surfactin molecules detected in the examined *Bacillus* strains.

The comparison of the results of the quantitative measurements is shown in Figure 2. Observing the diagram, it can be seen that none of the *B. megaterium* strains examined in this study produced surfactin at all. The extracted ferment broths of the five *B. velezensis* samples contained surfactin in the 2–5 mg/L concentration range, except for strain SZMC 24995 which bore the highest surfactin content among the examined samples by almost reaching 7 mg/L. The *B. atrophaeus* strain SZMC 24978 possessed biosurfactant production properties similar to most of the aforementioned strains. As a peculiar result in the case of *B. cereus*, the SZMC 24994 strain produced surfactins in one of the highest quantities among the examined samples, although, the ferment broth of the SZMC 25003 strain did not contain the observed lipopeptides. Results were similar in the case of the *B. pumilus* samples, in which strain SZMC 24991 did not produce the examined molecules, while strain SZMC 24987 possessed surfactins in the third highest concentration in its ferment broth. The quantity of biosurfactants in both *B. subtilis* strains were detected below 1 mg/L.

### 2.3. Identification of the Detected Surfactins

**Figure 3:**
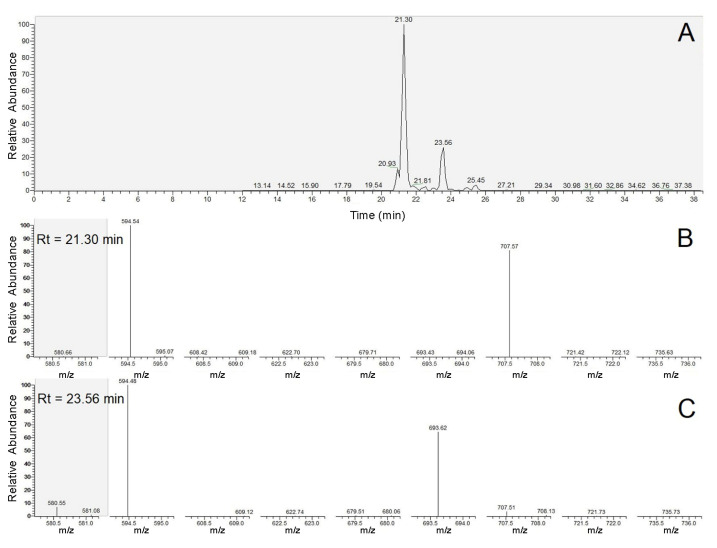
The extracted ion chromatogram (EIC) of *m*/*z* = 1016.7 of *B. velezensis* strain SZMC 24983 (**A**), and the MS^2^ spectra of the peaks at Rt = 21.30 min (**B**) and Rt = 23.56 min (**C**).

Identification of the different surfactin molecules was carried out based on the mass differences of the precursor ions and the y_6_ + H_2_O internal fragment ions as well as the *m*/*z* values of the overlapping peaks of y_6_ + H_2_O and y_6_b_6_ + H_2_O fragment ions. An example for the evaluation process is shown in Figure 3. Observing the MS/MS spectra of the two peaks, the first peak at the retention time of 21.30 min definitely belongs to a [Sur] variant, while the second one at Rt = 23.56 min marks the presence of a [Val7] surfactin molecule due to the corresponding *m*/*z* values of y_6_ + H_2_O and y_6_b_6_ + H_2_O fragment ions. Subtracting the *m*/*z* values of the y_6_ + H_2_O fragment ions from those of the sodiated precursor ions, their mass differences indicate the two homologues to be C12 and C13, respectively; thus, the two molecules can be identified as C12-[Sur] and C13-[Val7] simply from these acquired data.

**Figure 4:**
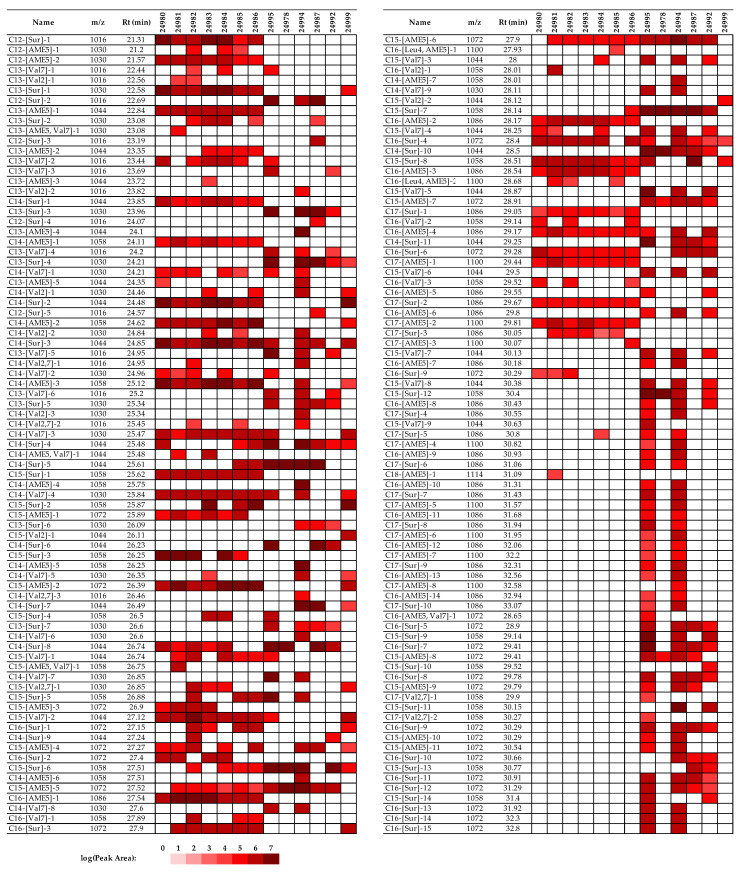
Relative amounts of the detected surfactin molecules of *B. atrophaeus* (SZMC 24978), *B. cereus* (SZMC 24994), *B. pumilus* (SZMC 24987), *B. subtilis* (SZMC 24992, SZMC 24999), and *B. velezensis* (SZMC 24980, SZMC 24981, SZMC 24982, SZMC 24983, SZMC 24984, SZMC 24985, SZMC 24986, SZMC 24995) strains.

By examining all of the peaks on the EICs in the case of every lipopeptide-producing sample, the detected surfactin variants and homologues were identified and listed in Figure 4 and in the Supplementary Material (Tables S1 and S2). There were 29 surfactin molecules with different structures, with most of them having multiple peaks with distinct retention times, suggesting changes in the branching of the fatty acid chains. As a result of the appearance of these specific peaks in vast numbers, 158 instances of them with particular retention times were detected altogether in all of the examined samples combined. Most of them occur in the *B. cereus* strain SZMC 24994 with 90 peaks of different surfactin molecules, while in evaluating the MS/MS spectra of the *B. atrophaeus* strain SZMC 24978, only 10 peaks were detected. While this number varies in a wide range in the other samples (10, 90, 36, 45, and 24 instances in SZMC 24978, 24994, 24987, 24992, and 24999, respectively), the strains of *B. velezensis* are consistent in that regard, resulting in a range from 41–55 instances with relatively high similarities with the only, rather peculiar, exception being the SZMC 24995 strain (Figure 4).

### 2.4. Comparison of the Surfactin Production Profiles

**Figure 5:**
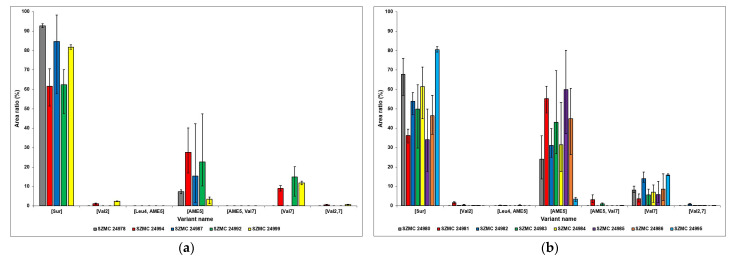
The ratios of all surfactin isoforms produced (**a**) by *B. atrophaeus* (SZMC 24978), *B. cereus* (SZMC 24994), *B. pumilus* (SZMC 24987), and *B. subtilis* (SZMC 24992, SZMC 24999) as well as (**b**) by *B. velezensis* (SZMC 24980, SZMC 24981, SZMC 24982, SZMC 24983, SZMC 24984, SZMC 24985, SZMC 24986, SZMC 24995) strains.

**Figure 6:**
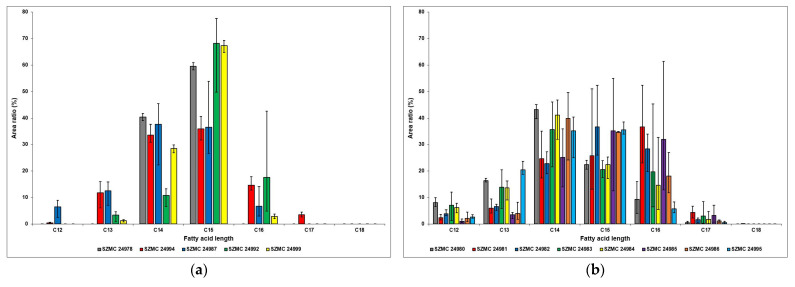
The ratios of all surfactin homologues produced (**a**) by *B. atrophaeus* (SZMC 24978), *B. cereus* (SZMC 24994), *B. pumilus* (SZMC 24987), and *B. subtilis* (SZMC 24992, SZMC 24999) as well as (**b**) by *B. velezensis* (SZMC 24980, SZMC 24981, SZMC 24982, SZMC 24983, SZMC 24984, SZMC 24985, SZMC 24986, SZMC 24995) strains.

After the successful identification of the different surfactin variants and homologues, their relative quantitative relations were examined by combining their respective integrated peak areas and comparing their area ratio percentages in diagrams (Figure 5 and Figure 6). Regarding the different variants, one main similarity can be observed between the strains of *B. atrophaeus*, *B. cereus*, *B. pumilus*, and *B. subtilis*, namely that the relative amount of [Sur] molecules is the most dominant, reaching over 60% in all cases and extending over 90% in strain SZMC 24978 (Figure 5). The variant with the second most dominant area ratio is [AME5], except for strain SZMC 24999, as it possesses [Val7] surfactins in higher amounts. A rather peculiar result is that the ferment broth of the *B. atrophaeus* strain SZMC 24978 contained only three molecules with different structures—C14-[Sur], C15-[Sur], and C15-[AME5]—although their total concentration has proven to be above average compared with the other examined *Bacillus* strains (Figure 2). The area ratios of the variants [Val2] and [Val2,7] are close to negligible, and the [Leu4, AME5] and [AME5, Val7] surfactins were not even detected in the samples of these four *Bacillus* species.

The surfactin variants with the highest relative amounts are also the [Sur], [AME5], and [Val7] isoforms in the case of the *B. velezensis* samples; however, the ratio of the first two are much closer together, except for strain SZMC 24995; the [AME5] variant even surpasses the [Sur] molecules in that regard in strains SZMC 24981 and SZMC 24985 (Figure 5). After those, the [Val7] isoforms are produced in the highest relative amounts, although only exceeding 10% in the SZMC 24982 and SZMC 24995 strains. The area ratios of the other surfactin variants are below 4% in all samples, not even reaching the 1% mark in most cases.

In observing the relative amounts of the different surfactin homologues in the strains of *B. atrophaeus*, *B. cereus*, *B. pumilus*, and *B. subtilis*, the diagram shows a common characteristic among these samples, which is the dominance of C14 and C15 molecules. The only exception is the SZMC 24992 strain, in which the C16 homologues have the second largest area ratio after the C15 surfactins (Figure 6). As a result of its unique production properties described above, no other homologues were detected in the ferment broth of the *B. atrophaeus* strain SZMC 24978. In the case of the *B. cereus* strain, all of the homologues were detected, except for the C18 surfactins, while the samples of *B. subtilis* are lacking the presence of C12, C17, and C18 molecules. However, both the SZMC 24992 and the SZMC 24999 strains produced C15 homologues in relative amounts of approximately 70%, which is the highest compared with all other samples.

Results of the *B. velezensis* strains show that the area ratios of C16 molecules are more proportionate to their C14 and C15 counterparts; in the SZMC 24981 strain, these were observed in the highest relative amounts (Figure 6). This is also the only sample in which C18 homologues were detected, although only in a mere 0.05% ratio, while all the other fatty acid chain lengths between 12 and 17 carbon atoms were present in all strains.

### 2.5. The Biocontrol Properties of the Examined Bacillus Isolates

Due to bacterial growth inhibitions, the clearance zones showed potential biocontrol activities of *Bacillus* isolates. Altogether, 16 of the 25 isolates exhibited such properties on pathogenic bacteria, with inhibition zones ranging from 1.0 to 16.67 mm. The *B. velezensis* strains were considered to have substantial potential for biocontrol, antagonizing against all test pathogens (Table 3). In a peculiar way, *E. amylovora* and *X. campestris* were significantly antagonized by a wide range of isolates. Accordingly, 36.0% of isolates exhibited notable activities against *E. amylovora*, with inhibition zone diameters of ≥5mm; moreover, 28.0% of those were ≥10mm. Subsequently, 28.0% of isolates exhibited activities against *X. campestris*, with inhibition zone diameters of ≥5mm and 4.0% of those ≥10mm. In addition, isolates slightly antagonized *A. tumefaciens*, *P. syringae*, and *E. carotovora* with lower activities. In all cases, the inhibitions were statistically significant based on ANOVA.

For the examination of biocontrol properties of the *Bacillus* isolates on pathogenic fungi, 14 of the 25 testing isolates were potent in the control of various phytopathogenic fungi, with inhibition rates ranging from 34.4% to 83.8%. The isolates of *B. subtilis* and *B. velezensis* showed significant activities in fungal growth inhibition. To sum up, 40%, 52%, 40%, 40%, and 36% of isolates inhibited more than 50% growth of *F. graminearum*, *B. cinerea*, *F. solani*, *F. culmorum*, and *C. gloeosporioides*, respectively. Furthermore, 44%, 12% and 4% of isolates effectively inhibited more than 70% growth of *B. cinerea*, *F. culmorum*, and *C. gloeosporioides*, respectively. In all cases, the inhibitions were statistically significant based on ANOVA (Table 4).

## 3. Discussion

The taxonomy identification relied on the Sherlock CAS method which revealed *Bacillus* isolates as *B. atrophaeus*, *B. cereus*, *B. megaterium*, *B. pumilus*, *B. subtilis*, and *B. velezensis*. Accordingly, FA composition has been diverse, drawing a distinction between *Bacillus* species as taxonomic biomarkers. As a rather peculiar result, FA compositions in *B. cereus*, *B. megaterium*, and *B. pumilus* have been divided into two distinguishable groups, named GC subgroup A and B. These species possess a higher content of branched-odd FAs, including 13:0 iso, 15:0 iso, 15:0 anteiso, 17:0 iso, and 17:0 anteiso, as common features of *Bacillus*’ taxonomy [26]. 

A combined SIM/SRM mass spectrometric method has also been developed, capable of performing quantitative measurements of the total surfactin concentration on the extracted ferment broths of the different strains and simultaneously identifying the different surfactin molecules based on the *m*/*z* values of their sodiated precursor ions and the first two internal fragment ions, whereby monitoring 80 mass transitions altogether. Out of the aforementioned 25 strains, 13 produced surfactins in average concentrations of 0.5–6.6 g/L while 12 samples, including the ferment broths of all *B. megaterium* strains, contained no surfactins whatsoever. As a result of our qualitative measurements, 29 surfactin molecules in total were identified, with 158 detected instances with different retention times, suggesting numerous variations of branching within their fatty acid chains apart from alterations in their chain lengths and amino acid sequences. In comparing the relative amounts of the different surfactin variants and homologues, the resulting data showed that the [Sur], [AME5], and [Val7] isoforms were the most dominant in all cases, while regarding the occurrence of surfactins with different fatty acid chain lengths, the C14–C16 molecules had the largest area ratios. Results supported the conclusions of our earlier studies stating that the appearance of a previously rarely encountered group of surfactins with methyl esterified aspartic acid in their fifth amino acid position could be encountered in considerable numbers, and the fatty acid chain lengths could vary between 12 and 18 carbon atoms.

The inhibitory results demonstrated that the *Bacillus* isolates have a broad range in the biocontrol potential against various phytopathogens. The use of bactericidal and fungicidal microbes as natural mechanisms may prevent the production losses in agriculture caused by phytopathogens and limit the effects of chemical pesticides and antibiotics on the environment and ecosystem [27]. Moreover, many rhizosphere-associated *Bacillus* exhibiting significant inhibitory activity towards phytopathogens have been reported in agreement that *Bacillus* species are ideal biocontrol candidates [27,28,29,30]. 

The present results exhibited the significantly effective biocontrol activity of *Bacillus* species against *E. amylovora*, *X. campestris*, *B. cinerea*, and *F. culmorum*. In addition, the strains displayed moderate effects on *P. syringae*, *E. carotovora*, *A. tumefaciens*, *F. graminearum*, *F. solani*, and *C. gloeosporioides*. Accordingly, it was determined that the strains belonging to the *B. subtilis* species complex, namely *B. atrophaeus*, *B. subtilis*, and *B. velezensis*, could inhibit test phytopathogens as effective biocontrol agents. Together with gene clusters encoding non-ribosomal synthesis of lipopeptides and polyketides, the *Bacillus* group has been reflected as a producer of diverse bioactive secondary metabolites [31]. The *Bacillus* species were described in 2005 [32] regarding plant–pathogen-inhibiting and plant–growth-promoting potentials [33,34]. The present investigation determined great potential, especially in the *B. velezensis* species which contains many gene clusters toward non-ribosomal synthesis of versatile metabolites [35]. 

Generally, it can be stated that the best surfactin producers are the members of the *B. velezensis* species because all studied members of this species produced these biosurfactants, which released in remarkably high variabilities (Figure 4). The number of the detected congeners varied in the range of 41–84 and 24–45 for *B. velezensis* and *B. subtilis*, respectively, while this number was 10, 90, and 30 for *B. atrophaeus*, *B. cereus*, and *B. pumilus*, respectively (Figure 4). 

In the fungicidal assays, the *B. velezensis* and *B. subtilis* isolates showed the highest inhibition rates against fungi, which typically ranged from 30–50%. Currently, there is no possibility to provide direct relationships between the surfactin productions and the biological activities based on the gathered result; however, in examining the surfactin profiles of these isolates to find common features, it can be concluded that strains of both species produced C14-[Sur] and C15-[Sur] in high amounts. Furthermore, the *B. velezensis* isolates showed the most effective antibacterial activities against the phytopathogen bacteria, producing C14-[Sur], C14-[Val7], C15-[Val7], C15-[Sur], and C16-[AME5] as shared features of their surfactin production. 

Based on our knowledge, our results have provided the most detailed surfactin characterisation of the *Bacillus* isolates, which can be extended in the future with the inclusion of novel strains belonging to other species or isolated from different locations/sources in order to achieve deeper insight into the surfactin production features of *Bacillus* strains.

## 4. Materials and Methods

### 4.1. Strains Maintenance 

The examined *Bacillus* strains were isolated from different vegetable rhizospheres (Table 1). The strains were derived from the Szeged Microbiology Collection (SZMC; www.szmc.hu (accessed on 16 January 2023)), maintained on nutrient agar (5 g/L peptone, 3 g/L yeast extract, 5 g/L NaCl, 15 g/L agar) slants, and stored at 4 °C. 

### 4.2. Nomenclature of Surfactin Variants

Surfactin variants were designated according to Grangemard et al. [36] and Bóka et al. [16]. Briefly, the first discovered surfactin sequence (Glu-Leu-Leu-Val-Asp-Leu-Leu) was denoted as [Sur], and any changes in the peptide sequence were indicated with the abbreviation and position of the altered amino acid, for example, [Val2], [Val7], and [Val2,7]. The esterified form of aspartic acid and glutamic acid at the side chain carboxyl group were abbreviated as AME and GME, respectively. As the applied mass spectrometric technique could not distinguish between the Leu and Ile isobaric residues, this sequence element was marked as Lxx in this paper. The amino acid residues present in the sequences of the surfactins are designated in general by AAn, the superscript ‘n’ indicating the position number of each amino acid from the N-terminal end of the peptide chain. Furthermore, the fragment ions on the MS^2^ spectra were designated according to the terminology published by Roepstorff and Fohlman [37], as well as Biemann [38], while the internal fragments of sodiated fragment ions were designated by the y_n_b_m_ nomenclature [16,39].

### 4.3. Culture Conditions and Sample Preparation for surfactin Analysis

For the surfactin production, a liquid ferment broth was applied according to Besson et al. [40] containing 10 g/L glucose, 5 g/L glutamic acid, 1 g/L KH_2_PO_4_, 1 g/L K_2_HPO_4_, 1 g/L KCl, 500 mg/L MgSO_4_ × 7 H_2_O, 5 mg/L FeSO_4_ × 7 H_2_O, and 160 µg/L CuSO_4_ × 5 H_2_O. Bacteria (5 × 107 cells) were inoculated into a 20 mL medium in 100 mL Erlenmeyer flasks followed by incubation on a rotary shaker at 120 rpm for five days at 25 °C.

The bacterial cells were separated from the ferment broths via centrifugation at 8000 rpm for 15 min at 4 °C. The pH of the supernatant was decreased to 2 with HCl, and the lipopeptides were precipitated overnight at 4 °C. The pellets were collected by centrifugation (8000 rpm, 15 min, 4 °C) and resolved in 1 mL methanol [23].

All chemicals and reagents mentioned above were AR purity and were purchased from Molar Chemicals Ltd. (Budapest, Hungary).

### 4.4. Identification of Bacillus Isolates by Fatty Acid Methyl Ester (FAME) Analysis

The MIDI Sherlock^®^ Microbial Identification System (MIS, Microbial ID Inc., Newark, NJ, USA) was applied for the identification. The composition of whole-cell fatty acids was determined by the Sherlock CAS Software operating on a gas chromatography platform, Shimadzu’s GC-2010/2030, equipped with an HP-Ultra 2, 25 m × 0.2 mm × 0.33 µm thickness fused silica capillary column (Agilent, Santa Clara, CA, USA) as a stationary phase [25,41]. Briefly, the sample processing was prepared following The SherlockTM Operating CAS Manual. The bacteria were cultured on Trypticase Soy Broth Agar (Becton, Dickinson and Company, Sparks, NV, USA) at 28 °C for 24 ± 2 h. Then, cells were harvested, saponificated, methylated, and extracted producing total FAMEs. The whole-cell FAME profiles with database were analysed by the method RTSBA6 and the library RTSBA6 and RTSBA7. In the method, injector and detector temperatures were 250 °C and 300 °C, respectively. Carrier gas was hydrogen at a flow rate of 1.48 mL/min, while the detector gases were nitrogen (make up), oxygen, and hydrogen with the flows of 30, 30, and 350 mL/min, respectively. Samples were introduced in an injection volume of 2 µL in split mode with a 40:1 split ratio. The oven program started at 168.1 °C, which ramped up to 291 °C at 28 °C per min and then up to 300 °C at 60 °C per min, holding at this temperature for 1.50 min. The total column oven program time was 6.04 min. The 1300-C rapid calibration standard mix (Microbial ID Inc., Newark, DE, USA) was used for RT calibration and system suitability purposes as well as for the fine tuning of the pressure and temperature parameters at the system setup.

The *B. subtilis* ATCC 6633 and pure hexane were considered as the positive and negative control, respectively.

### 4.5. Analytical Parameters

The HPLC-HESI-MS/MS examinations of the different surfactin molecules were carried out based on the work of Büchner et al. [42]. The applied instrument was a Nexera XR HPLC system containing a DGU-20A5R degasser, an LC-20ADXR pump, a SIL-20AXR autosampler and a CTO-10ASVP column oven (Shimadzu Corporation, Kyoto, Japan), coupled with a TSQ Quantum Access triple quadrupole mass spectrometer (Thermo Scientific, Waltham, MA, USA).

The gradient solvent delivery system consisted of two solvents: A was H_2_O, and B was a mixture of acetonitrile/methanol (1:1, *v*/*v* %). Both solvents were supplemented with 0.1% acetic acid. The applied reverse phase gradient elution time program was the following: 5% eluent B for 2 min, increased to 80% in the following 2 min, and then gradually raised to 95% for 24 min. This rate was held for 9 min and then dropped to 5% in 0.5 min, followed by a 5 min long equilibration stage, ending the run of 38.5 min in total. The flow rates were 0.2 mL/min and the column heater temperature was 30 °C. The applied column was a Gemini-NX (3µ, C18, 150 × 2 mm). The injection volume was 10 µL.

Both the quantitative and qualitative measurements were carried out using a combined method in selected ion monitoring (SIM) and single reaction monitoring (SRM) modes running in parallel, with a heated electrospray ionization (HESI) ion source and in positive polarity. The spray voltage was +4000 V; the vaporiser temperature was 285 °C; the capillary temperature was 350 °C; the sheath gas pressure was 10 psi; and the auxiliary gas pressure was 15 psi. The SRM mode analyses for the identification of the different surfactin variants were performed with a collision energy of 60 V and a collision gas pressure of 1 mTorr. The *m*/*z* values of sodiated surfactin molecules were set as parent ion masses (*m*/*z* 1016.7, 1030.7, 1044.7, 1058.7, 1072.7, 1086.7, 1100.7, 1114.7) and for every parent ion, the first two internal fragment ions of every natural surfactin variant were set as a daughter ion (*m*/*z* 580.7, 594.7, 608.7, 622.7, 679.7, 693.7, 707.7, 721.7, 735.7). The SIM mode measurements were also used to examine the parent ion *m*/*z* values listed above. With these two modes, 80 different mass transitions ran in parallel for a total scan time of 0.11 sec/scan. The instrument control and the data processing were performed using the TraceFinder General Quan 4.1 software (Thermo Fisher Scientific, Waltham, MA, USA) while the Xcalibur software v. 4.0 (Thermo Fisher Scientific, Waltham, MA, USA) was applied for the spectral examinations.

The standard used for the quantification was surfactin (S3523) from Sigma-Aldrich (Budapest, Hungary). All chemicals used as eluents and for sample preparation are HPLC-MS purity and were purchased from VWR International Ltd. (Debrecen, Hungary).

### 4.6. Inhibition Assays on Phytopathogen Microorganisms

The tests were determined by an agar diffusion technique [30] using the yeast extract–glucose medium (YEG) (glucose 0.2%, yeast extract 0.2%, bacto-agar 2%; purchased from VWR International Ltd. (Debrecen, Hungary)).

The following bacteria were prepared in YEG broth overnight: *Pseudomonas syringae* SZMC 16160, *Erwinia amylovora* SZMC 21402, *Erwinia carotovora* SZMC 6190, *Xanthomonas campestris* SZMC 6182, *Agrobacteria tumefaciens* SZMC 14554 and *Bacillus* isolates. A quantity of 200 µL of phytopathogenic suspension (~5 × 107 CFU/mL) was spread on YEG agar, and then 6 mm diameter paper discs with 5 µL of *Bacillus* suspension (~2.5 × 107 CFU/mL) were put in place. The antibacterial effects were evaluated by measuring inhibition zones after 1–2 days. Three replicates were conducted for each experiment.

The following fungi were prepared on PDA plates: *Fusarium graminearum* SZMC 11030, *Botrytis cinerea* SZMC 21047, *Fusarium solani* SZMC 16084, *Fusarium culmorum* SZMC 11039, and *Colletotrichum gloeosporioides* SZMC 16087. In addition, *Bacillus* isolates were prepared in YEG broth overnight. Subsequently, 6 mm in diameter paper discs with 5 µL of *Bacillus* suspension (~2.5 × 10^7^ CFU/mL) and 6 mm in diameter mycelia from cultured fungi were inoculated on YEG agar by using a direct dual culture method with a 3 cm spacing distance. The control test was performed without *Bacillus*. Three replicates were conducted for each experiment. The fungicidal activity was determined by the values of the inhibition rate (%) after the 7-day incubation.

The rate of inhibition was measured using the formula: The inhibition rate (%) = ((diameter of control − diameter of treatment)/diameter of control) × 100%.

### 4.7. Statistical Analysis

Data were displayed as Mean ± SD. Analysis of variance (ANOVA) was run using the R package. Values with *p* < 0.05 were considered statistically significant.

## 5. Conclusions

In this study, 25 *Bacillus* strains in total isolated from vegetable rhizospheres were identified by their FAME profiles and were tested against phytopathogen bacteria and fungi. Their surfactin production profiles were also examined in detail by a fast and easily evaluable HPLC-HESI-MS/MS method in SIM/SRM mode, differentiating 158 surfactin variants from the ferment broths. 

Based on our knowledge, our results report the most comprehensive surfactin profiling, which could promote the further targeted selection of strains and cultivation conditions to produce certain surfactin molecules responsible mainly for biological effects. 

## Figures and Tables

**Table 1:** List of the identified *Bacillus* isolates.

Culture Collection Number ^1^	Source	Identification
SZMC 24978	Totovo selo, Serbia, soil sample, tomato	*B. atrophaeus*
SZMC 24979	Szeged, Hungary, soil sample, tomato	*B. megaterium*
SZMC 24980	Szeged, Hungary soil sample, pepper	*B. velezensis*
SZMC 24981	Szeged, Hungary soil sample, pepper	*B. velezensis*
SZMC 24982	Szeged, Hungary soil sample, pepper	*B. velezensis*
SZMC 24983	Szeged, Hungary soil sample, pepper	*B. velezensis*
SZMC 24984	Szeged, Hungary soil sample, pepper	*B. velezensis*
SZMC 24985	Szeged, Hungary soil sample, pepper	*B. velezensis*
SZMC 24986	Szeged, Hungary soil sample, tomato	*B. velezensis*
SZMC 24987	Cantavir, Serbia, tomato	*B. pumilus*
SZMC 24988	Szeged, Hungary soil sample, tomato	*B. megaterium*
SZMC 24990	Szeged, Hungary soil sample, tomato	*B. megaterium*
SZMC 24991	Szeged, Hungary soil sample, tomato	*B. pumilus*
SZMC 24992	Cantavir, Serbia, pepper	*B. subtilis*
SZMC 24993	Szeged, Hungary soil sample, pepper	*B. megaterium*
SZMC 24994	Cantavir, Serbia, tomato	*B. cereus*
SZMC 24995	Cantavir, Serbia, tomato	*B. velezensis*
SZMC 24997	Szeged, Hungary soil sample, carrot	*B. megaterium*
SZMC 24998	Szeged, Hungary soil sample, carrot	*B. megaterium*
SZMC 24999	Szeged, Hungary soil sample, carrot	*B. subtilis*
SZMC 25000	Szeged, Hungary soil sample, paprika	*B. megaterium*
SZMC 25001	Madaras, Hungary, pepper	*B. megaterium*
SZMC 25002	Szeged, Hungary soil sample, paprika	*B. megaterium*
SZMC 25003	Szeged, Hungary soil sample, paprika	*B. cereus*
SZMC 25004	Szeged, Hungary soil sample, sweet potato	*B. megaterium*

^1^ SZMC—Szeged Microbiology Collection.

**Table 2:** Cellular fatty acid compositions (%) of the identified species.

Features	*B. atrophaeus*	*B. cereus* (GC-A) ^1^	*B. megaterium* (GC-A) ^1^	*B. pumilus* (GC-B) ^1^	*B. subtilis*	*B. velezensis*
13:0 iso	-	9.69	-	0.50	-	0.93
14:0 iso	1.37	3.61	4.31	0.79	0.85	1.05
14:0	-	2.91	1.72	1.23	-	3.04
15:0 iso	13.53	34.78	38.02	50.83	24.24	30.25
15:0 anteiso	46.28	3.93	41.56	23.48	39.18	32.54
16:0 iso	4.11	5.56	0.62	1.44	2.37	1.56
16:1 w11c	1.82	-	3.85	1.88	1.74	1.71
16:0	2.97	4.97	2.70	4.15	3.39	13.00
17:1 iso w10c	1.81	4.69	0.57	2.26	2.44	0.82
17:0 iso	6.29	9.00	1.86	8.12	10.94	8.18
17:1 iso w5c	-	4.50	-	-	-	-
17:0 anteiso	17.92	0.96	3.25	4.56	12.34	5.43

^1^ GC-A: GC subgroup A and GC-B: GC subgroup B.

**Table 3:** Inhibition of bacterial pathogens by isolates.

Species	Strain Number	*P. syringae*	*E. amylovora*	*E. carotovora*	*X. campestris*	*A. tumefaciens*
		Inhibition Zones *				
*B. atrophaeus*	SZMC 24978	-	++	+	+	+
*B. cereus*	SZMC 25003	-	+	-	-	-
*B. megaterium*	SZMC 24979	-	+++	+	++	+
	SZMC 24988	-	-	-	-	+
	SZMC 25000	-	+	-	-	-
*B. pumilus*	SZMC 24991	-	-	-	-	+
*B. subtilis*	SZMC 24992	-	+	+	+	+
	SZMC 24999	-	+	-	-	-
*B. velezensis*	SZMC 24980	+	++	+	++	+
	SZMC 24981	+	+++	+	++	+
	SZMC 24982	+	+++	+	++	+
	SZMC 24983	+	+++	+	++	+
	SZMC 24984	+	+++	+	+++	+
	SZMC 24985	-	+++	+	++	+
	SZMC 24986	+	+++	-	+	+
	SZMC 24995	+	+	+	-	+

* Inhibition zone: no inhibition (-), 1–5 mm (+), 5.1–10 mm (++), and >10 mm (+++).

**Table 4:** Inhibition of bacterial pathogens by isolates.

Species	Strain Number	*F. graminearum*	*B. cinerea*	*F. solani*	*F. culmorum*	*C. gloeosporioides*
		Inhibition Rates *				
*B. atrophaeus*	SZMC 24978	-	+++	-	-	-
*B. cereus*	SZMC 25003	-	++	-	-	+
*B. megaterium*	SZMC 24979	++	-	++	-	-
*B. pumilus*	SZMC 24987	-	++	-	-	-
*B. subtilis*	SZMC 24992	++	+++	++	++	-
	SZMC 24999	++	+++	++	++	+++
*B. velezensis*	SZMC 24980	+	+++	++	++	++
	SZMC 24981	++	+++	++	+++	++
	SZMC 24982	++	+++	++	++	++
	SZMC 24983	++	+++	+	+++	++
	SZMC 24984	++	+++	++	++	++
	SZMC 24985	++	+++	++	++	++
	SZMC 24986	++	+++	++	++	++
	SZMC 24995	++	+++	++	+++	++

* Inhibition rate: no inhibition (-), 30–50% (+), 51–70% (++), and >70% (+++).

## Data Availability

Not applicable.

## Supplementary Materials

The following supporting information can be downloaded at: https://www.mdpi.com/article/10.3390/molecules28031172/s1, Table S1: Relative amounts of the detected surfactin molecules of *B. atrophaeus* (SZMC 24978), *B. cereus* (SZMC 24994), *B. pumilus* (SZMC 24987) and *B. subtilis* (SZMC 24992, SZMC 24999); Table S2: Relative amounts of the detected surfactin molecules of *B. velezensis* strains.
