# Supplementary material for: Identifications of Surfactin-Type Biosurfactants Produced by Bacillus Species Isolated from Rhizosphere of Vegetables
Source: Molecules. 2023 Jan 25;28(3):1172. doi: 10.3390/molecules28031172 (PMC9919572; doi:10.3390/molecules28031172)
Supplement: Supplementary file 1 [file molecules-28-01172-s001.zip › molecules-2170649-supplementary.pdf]

molecules

Identifications of surfactin-type biosurfactans produced by *Bacillus* species isolated from rhizosphere of vegetables

Attila Bartal [1], Thu Huynh [1,2,3], Anita Kecskeméti [2], Mónika Vörös [1], Orsolya Kedves [1], Allaga Henrietta[1], László Kredics [1], Csaba Vágvölgyi [1] and András Szekeres [1,*]

[1] Department of Microbiology, Faculty of Science and Informatics, University of Szeged, Közép Fasor 52, H-6726 Szeged, Hungary
[2] Department of Biotechnology, Faculty of Chemical Engineering, Ho Chi Minh University of Technology (HCMUT), 268 Ly Thuong Kiet Street, District 10, Ho Chi Minh City 72607, Vietnam
[3] Vietnam National University Ho Chi Minh City, Linh Trung Ward, Thu Duc District, Ho Chi Minh City 71351, Vietnam

* Correspondence: szandras@bio.u-szeged.hu; Tel.: +36-62-544516

Table S1. Relative amounts of the detected surfactin molecules of *B. atrophaeus* (SZMC 24978), *B. cereus* (SZMC 24994), *B. pumilus* (SZMC 24987) and *B. subtilis* (SZMC 24992, SZMC 24999).

**Name**	**m/z**	**Rt (min)**	**24978**	**24994**	**24987**	**24992**	**24999**
			**Average peak area***				
C12-[Sur]-1	1016	21.31	-	-	-	-	-
C12-[AME5]-2	1030	21.57	-	-	-	-	-
C13-[Val7]-1	1016	22.44	-	-	-	-	-
C13-[Sur]-1	1030	22.58	-	-	-	-	70028 (49.47)
C12-[Sur]-2	1016	22.69	-	244674 (86.44)	1611151 (74.17)	-	-
C13-[AME5]-1	1044	22.84	-	-	-	-	-
C13-[Sur]-2	1030	23.08	-	-	4262 (5.82)	-	-
C12-[Sur]-3	1016	23.19	-	-	510456 (82.71)	-	-
C13-[Val7]-2	1016	23.44	-	-	-	-	-
C13-[Val7]-3	1016	23.69	-	-	-	1969 (14.52)	-
C13-[Val2]-2	1016	23.82	-	49473 (97.86)	-	-	-
C14-[Sur]-1	1044	23.85	-	-	-	-	-
C13-[Sur]-3	1030	23.96	-	1464396 (75.95)	1655488 (71.68)	83341 (107.81)	-
C12-[Sur]-4	1016	24.07	-	-	52132 (74.74)	-	-
C13-[AME5]-4	1044	24.10	-	1207773 (89.24)	-	-	-
C14-[AME5]-1	1058	24.11	-	-	-	-	-
C13-[Val7]-4	1016	24.20	-	44856 (110.36)	-	3945 (70.05)	-
C13-[Sur]-4	1030	24.21	-	2098845 (79.87)	2234097 (73.85)	60910 (102.26)	3284 (82.01)
C14-[Val7]-1	1030	24.21	-	57756 (68.94)	-	-	-
C13-[AME5]-5	1044	24.35	-	542422 (89.72)	-	-	-
C14-[Val2]-1	1030	24.46	-	184008 (79.85)	-	-	11053 (31.70)
C14-[Sur]-2	1044	24.48	-	-	-	-	1195556 (17.60)
C12-[Sur]-5	1016	24.57	-	-	15240 (111.19)	-	-
C14-[AME5]-2	1058	24.62	-	-	-	-	45189 (82.55)
C14-[Val2]-2	1030	24.84	-	105787 (125.18)	-	-	-
C14-[Sur]-3	1044	24.85	-	311594 (69.42)	106542 (124.81)	-	221627 (25.57)

C13-[Val7]-5	1016	24.95	-	382917 (72.97)	-	28179 (86.93)	-
C14-[Val2,7]-1	1016	24.95	-	181988 (96.05)	-	-	-
C14-[Val7]-2	1030	24.96	-	-	-	-	-
C14-[AME5]-3	1058	25.12	-	122001 (41.41)	-	-	3379 (77.03)
C13-[Val7]-6	1016	25.20	-	161817 (119.14)	-	33006 (88.58)	-
C13-[Sur]-5	1030	25.34	-	193746 (78.99)	133001 (76.48)	15745 (59.16)	-
C14-[Val2]-3	1030	25.34	-	241083 (67.34)	-	-	-
C14-[Val2,7]-2	1016	25.45	-	32042 (87.39)	-	-	-
C14-[Val7]-3	1030	25.47	-	-	-	-	132910 (14.50)
C14-[Sur]-4	1044	25.48	-	2916937 (78.10)	129503 (43.92)	19225 (103.68)	15238 (100.10)
C14-[Sur]-5	1044	25.61	4060202 (16.37)	1765098 (35.77)	3135825 (80.19)	-	-
C15-[Sur]-1	1058	25.62	-	-	-	-	-
C14-[AME5]-4	1058	25.75	-	1070542 (54.36)	-	-	-
C14-[Val7]-4	1030	25.84	-	113135 (37.38)	-	-	20269 (45.70)
C15-[Sur]-2	1058	25.87	-	-	-	-	2962203 (14.00)
C15-[AME5]-1	1072	25.89	-	-	-	-	-
C13-[Sur]-6	1030	26.09	-	34004 (75.66)	23710 (77.44)	5178 (9.67)	-
C15-[Val2]-1	1044	26.11	-	-	-	-	107097 (16.15)
C14-[Sur]-6	1044	26.23	-	-	3028048 (34.41)	423783 (67.12)	-
C15-[Sur]-3	1058	26.25	-	-	-	-	-
C14-[AME5]-5	1058	26.25	-	1711555 (43.22)	-	-	-
C14-[Val7]-5	1030	26.35	-	133681 (45.23)	-	-	3972 (137.77)
C15-[AME5]-2	1072	26.39	-	-	-	-	155937 (59.52)
C14-[Val2,7]-3	1016	26.46	-	67538 (80.01)	-	-	-
C14-[Sur]-7	1044	26.49	-	3558476 (50.14)	2415026 (42.27)	-	9723 (43.15)
C15-[Sur]-4	1058	26.50	-	-	-	-	-
C13-[Sur]-7	1030	26.60	-	96398 (75.94)	17969 (74.04)	9236 (41.41)	-
C14-[Val7]-6	1030	26.60	-	515931 (5.31)	-	-	-

C14-[Sur]-8	1044	26.74	2315743 (33.85)	-	5327045 (0.91)	175680 (75.90)	-
C15-[Val7]-1	1044	26.74	-	-	-	-	-
C14-[Val7]-7	1030	26.85	-	534119 (71.06)	-	-	-
C15-[Val2,7]-1	1030	26.85	-	-	-	-	42682 (29.76)
C15-[Sur]-5	1058	26.88	-	617787 (45.51)	-	-	-
C15-[AME5]-3	1072	26.90	-	-	-	-	-
C15-[Val7]-2	1044	27.12	-	-	-	-	523027 (10.89)
C16-[Sur]-1	1072	27.15	-	-	-	-	25832 (89.29)
C14-[Sur]-9	1044	27.24	-	-	-	40287 (94.58)	-
C15-[AME5]-4	1072	27.27	-	434710 (89.75)	350114 (165.27)	-	7415 (44.91)
C16-[Sur]-2	1072	27.40	-	-	-	-	-
C15-[Sur]-6	1058	27.51	4483465 (8.02)	2735939 (67.98)	-	1491920 (61.57)	46177 (17.60)
C14-[AME5]-6	1058	27.51	-	174272 (56.97)	-	-	-
C15-[AME5]-5	1072	27.52	1177659 (20.74)	1070151 (45.77)	699622 (110.90)	194843 (22.85)	-
C16-[AME5]-1	1086	27.54	-	-	-	-	-
C14-[Val7]-8	1030	27.60	-	175227 (36.15)	-	-	-
C16-[Sur]-3	1072	27.90	-	-	-	-	145048 (37.36)
C15-[AME5]-6	1072	27.90	138555 (4.15)	1226541 (22.72)	622892 (118.96)	410096 (25.75)	-
C16-[Leu4, AME5]-1	1100	27.93	-	-	-	-	-
C15-[Val7]-3	1044	28.00	-	239859 (69.50)	-	35404 (49.43)	-
C14-[AME5]-7	1058	28.01	-	160587 (47.54)	-	-	-
C14-[Val7]-9	1030	28.11	-	206471 (48.82)	-	-	-
C15-[Val2]-2	1044	28.12	-	-	-	-	17997 (27.00)
C15-[Sur]-7	1058	28.14	3907059 (13.92)	2926184 (50.96)	4017466 (73.73)	729624 (90.22)	-
C16-[AME5]-2	1086	28.17	-	-	-	-	-
C15-[Val7]-4	1044	28.25	-	460252 (71.46)	-	78860 (73.94)	-
C16-[Sur]-4	1072	28.40	-	460030 (34.15)	43142 (128.76)	4367 (114.76)	6797 (123.19)
C14-[Sur]-10	1044	28.50	1365548 (54.07)	842750 (71.07)	282128 (100.70)	36064 (91.07)	-

C15-[Sur]-8	1058	28.51	-	-	2730548 (73.99)	-	43170 (41.30)
C16-[AME5]-3	1086	28.54	-	-	-	-	-
C15-[Val7]-5	1044	28.87	-	814811 (83.01)	-	608155 (76.90)	-
C15-[AME5]-7	1072	28.91	59171 (27.49)	116653 (27.73)	190820 (3.89)	51788 (35.05)	-
C17-[Sur]-1	1086	29.05	-	-	-	-	-
C16-[Val7]-2	1058	29.14	-	-	-	-	-
C16-[AME5]-4	1086	29.17	-	407082 (66.92)	-	178815 (153.28)	-
C14-[Sur]-11	1044	29.25	-	320740 (54.07)	588269 (86.33)	15749 (68.18)	-
C16-[Sur]-6	1072	29.28	-	413457 (28.79)	158559 (86.72)	108948 (23.07)	-
C17-[AME5]-1	1100	29.44	-	-	-	-	-
C15-[Val7]-6	1044	29.50	-	254964 (37.97)	-	163381 (75.50)	-
C16-[Val7]-3	1058	29.52	-	-	-	-	-
C16-[AME5]-5	1086	29.55	-	-	-	61212 (56.54)	-
C17-[Sur]-2	1086	29.67	-	-	-	-	-
C16-[AME5]-6	1086	29.80	-	452008 (25.11)	-	47316 (127.83)	-
C17-[AME5]-2	1100	29.81	-	-	-	-	-
C15-[Val7]-7	1044	30.13	-	195075 (59.77)	-	52265 (76.18)	-
C16-[AME5]-7	1086	30.18	-	208984 (64.91)	-	-	-
C15-[Val7]-8	1044	30.38	-	173314 (80.85)	-	31070 (86.77)	-
C15-[Sur]-12	1058	30.40	1614266 (25.34)	515349 (75.30)	-	71447 (69.81)	-
C16-[AME5]-8	1086	30.43	-	160957 (52.28)	-	20902 (126.51)	-
C17-[Sur]-4	1086	30.55	-	115161 (77.46)	-	-	-
C15-[Val7]-9	1044	30.63	-	-	-	-	-
C17-[Sur]-5	1086	30.80	-	89241 (71.90)	-	-	-
C17-[AME5]-4	1100	30.82	-	100531 (68.42)	-	-	-
C16-[AME5]-9	1086	30.93	-	153815 (24.48)	-	-	-
C17-[Sur]-6	1086	31.06	-	94743 (110.31)	-	-	-
C16-[AME5]-10	1086	31.31	-	137260 (8.08)	-	-	-
C17-[Sur]-7	1086	31.43	-	148530 (57.98)	-	-	-

C17-[AME5]-5	1100	31.57	-	176017 (34.75)	-	-	-
C16-[AME5]-11	1086	31.68	-	96553 (86.02)	-	-	-
C17-[Sur]-8	1086	31.94	-	78485 (8.45)	-	-	-
C17-[AME5]-6	1100	31.95	-	113243 (60.29)	-	-	-
C16-[AME5]-12	1086	32.06	-	50737 (46.18)	-	-	-
C17-[AME5]-7	1100	32.20	-	35041 (57.59)	-	-	-
C17-[Sur]-9	1086	32.31	-	113350 (31.72)	-	-	-
C16-[AME5]-13	1086	32.56	-	122488 (53.02)	-	-	-
C17-[AME5]-8	1100	32.58	-	51107 (80.20)	-	-	-
C16-[AME5]-14	1086	32.94	-	73416 (8.97)	-	-	-
C17-[Sur]-10	1086	33.07	-	379520 (57.37)	-	-	-
C16-[AME5, Val7]-1	1072	28.65	-	-	-	-	-
C16-[Sur]-5	1072	28.90	-	523017 (32.52)	225423 (59.63)	71311 (62.75)	-
C15-[Sur]-9	1058	29.14	-	947400 (35.78)	-	139273 (65.37)	-
C16-[Sur]-7	1072	29.41	-	349260 (13.62)	117412 (82.49)	72252 (77.08)	-
C15-[AME5]-8	1072	29.41	12004 (47.05)	184535 (24.24)	75539 (19.29)	-	-
C15-[Sur]-10	1058	29.52	-	-	-	68277 (3.73)	-
C16-[Sur]-8	1072	29.78	-	924021 (83.93)	219479 (25.18)	67028 (42.38)	-
C15-[AME5]-9	1072	29.79	-	166501 (64.48)	63110 (124.94)	-	-
C17-[Val2,7]-1	1058	29.90	-	-	-	-	-
C15-[Sur]-11	1058	30.15	-	1980365 (71.22)	-	142447 (76.46)	-
C17-[Val2,7]-2	1058	30.27	-	-	-	-	-
C16-[Sur]-9	1072	30.29	-	400641 (99.74)	295975 (65.40)	47483 (45.30)	-
C15-[AME5]-10	1072	30.29	-	212449 (60.57)	-	-	-
C15-[AME5]-11	1072	30.54	-	348567 (61.65)	-	-	-
C16-[Sur]-10	1072	30.66	-	323834 (49.51)	91983 (19.35)	18054 (57.77)	-
C15-[Sur]-13	1058	30.77	-	-	210787 (92.01)	40726 (94.66)	-
C16-[Sur]-11	1072	30.91	-	379216 (56.57)	129776 (59.69)	5131 (21.74)	-
C16-[Sur]-12	1072	31.29	-	466617 (74.88)	47508 (32.04)	6739 (76.42)	-

C15-[Sur]-14	1058	31.40	-	-	-	48267 (55.18)	-
C16-[Sur]-13	1072	31.92	-	138640 (52.66)	-	-	-
C16-[Sur]-14	1072	32.30	-	285593 (64.29)	-	-	-
C16-[Sur]-15	1072	32.80	-	199251 (78.07)	-	-	-

* RSD% are in brackets.

Table S2. Relative amounts of the detected surfactin molecules of *B. velezensis* strains.

Name	m/z	Rt (min)	24980	24981	24982	24983	24984	24985	24986	24995
			Average peak area*							
C12-[Sur]-1	1016	21.31	1590715 (118.01)	156806 (63.41)	444016 (43.25)	1085450 (125.31)	1790471 (78.95)	57623 (153.74)	146351 (65.34)	-
C12-[AME5]-1	1030	21.20	-	-	12455 (48.20)	-	62393 (72.96)	3338 (58.34)	-	-
C12-[AME5]-2	1030	21.57	210231 (77.66)	140353 (32.14)	112138 (59.84)	259186 (96.12)	264023 (71.09)	30830 (65.77)	45735 (55.34)	-
C13-[Val7]-1	1016	22.44	-	-	7211 (125.63)	-	23795 (81.50)	-	-	32589 (128.95)
C13-[Val2]-1	1016	22.56	-	2214 (105.36)	5328 (94.08)	-	-	-	-	-
C13-[Sur]-1	1030	22.58	2516155 (108.73)	311710 (56.52)	539815 (18.89)	1511427 (123.04)	2766554 (77.03)	104372 (129.81)	214155 (66.38)	-
C12-[Sur]-2	1016	22.69	-	-	-	-	-	-	-	1765629 (54.49)
C13-[AME5]-1	1044	22.84	518681 (67.01)	326333 (33.47)	232220 (10.97)	594768 (86.40)	940974 (71.57)	128476 (36.47)	118896 (4.46)	-
C13-[Sur]-2	1030	23.08	-	-	88818 (57.75)	241642 (141.75)	402243 (74.39)	-	8112 (80.51)	-
C13-[AME5, Val7]-1	1030	23.08	-	53246 (3.86)	-	-	-	-	-	-
C13-[AME5]-2	1044	23.35	-	-	-	69081 (77.49)	79420 (101.85)	13209 (132.08)	33664 (60.03)	-
C13-[Val7]-2	1016	23.44	282756 (131.36)	-	66122 (60.43)	212141 (134.58)	397961 (83.90)	20532 (138.59)	-	11084 (52.73)
C13-[Val7]-3	1016	23.69	-	-	-	-	-	-	-	185384 (44.66)
C13-[AME5]-3	1044	23.72	-	-	-	9522 (75.86)	-	-	-	-
C14-[Sur]-1	1044	23.85	120329 (73.09)	72318 (47.51)	50269 (67.97)	178708 (97.69)	273981 (69.74)	22081 (36.81)	30248 (63.35)	-
C13-[Sur]-3	1030	23.96	-	-	-	-	-	-	-	5537116 (26.88)
C14-[AME5]-1	1058	24.11	84574 (83.38)	163882 (22.88)	79009 (32.18)	244592 (90.88)	44144 (8.11)	54175 (79.45)	43048 (112.38)	-
C13-[Val7]-4	1016	24.20	-	-	-	-	-	-	-	140557 (22.77)
C13-[Sur]-4	1030	24.21	-	-	-	-	-	-	-	6454147 (83.28)
C14-[Val7]-1	1030	24.21	54472 (110.53)	19461 (121.86)	16567 (81.04)	-	55613 (87.73)	3447 (122.95)	-	48928 (35.50)
C13-[AME5]-5	1044	24.35	4000 (52.05)	-	-	-	-	-	-	-
C14-[Val2]-1	1030	24.46	-	-	-	64472 (154.55)	-	-	29374 (100.60)	-
C14-[Sur]-2	1044	24.48	2459202 (112.39)	328629 (50.55)	696443 (32.89)	1817146 (130.34)	3730982 (75.36)	154418 (115.14)	188080 (109.46)	-

C14-[AME5]-2	1058	24.62	528929 (68.59)	575795 (17.48)	153305 (51.60)	699775 (95.87)	1165064 (32.29)	338778 (77.96)	1013100 (76.97)	-
C14-[Val2]-2	1030	24.84	-	-	-	48574 (140.68)	-	5190 (55.68)	-	-
C14-[Sur]-3	1044	24.85	3135590 (113.26)	565057 (51.74)	766815 (19.13)	2204054 (136.84)	4706287 (80.06)	329902 (94.79)	1681822 (80.58)	235956 (60.11)
C13-[Val7]-5	1016	24.95	-	-	-	-	-	-	-	1427024 (87.94)
C14-[Val2,7]-1	1016	24.95	-	-	10389 (52.06)	-	-	-	-	-
C14-[Val7]-2	1030	24.96	29163 (76.35)	8523 (12.63)	27759 (29.44)	-	44594 (72.79)	-	-	55428 (157.56)
C14-[AME5]-3	1058	25.12	1156831 (70.18)	837912 (57.93)	628661 (42.87)	1457570 (109.19)	1921755 (70.15)	907250 (100.34)	1990704 (74.32)	-
C13-[Val7]-6	1016	25.20	-	-	-	-	-	-	-	653064 (21.40)
C13-[Sur]-5	1030	25.34	-	-	-	-	-	-	-	622664 (48.26)
C14-[Val2,7]-2	1016	25.45	-	-	4512 (87.68)	-	-	6917 (166.95)	-	-
C14-[Val7]-3	1030	25.47	438194 (113.51)	40513 (69.73)	115958 (35.74)	349806 (140.28)	700496 (83.59)	130082 (136.62)	110470 (98.10)	131235 (21.99)
C14-[Sur]-4	1044	25.48	189248 (76.46)	-	-	-	-	91964 (87.17)	105382 (90.26)	1458018 (137.16)
C14-[AME5, Val7]-1	1044	25.48	-	97729 (63.70)	-	196772 (130.03)	-	-	-	-
C14-[Sur]-5	1044	25.61	-	-	-	-	-	105183 (101.80)	104740 (79.08)	6033697 (23.99)
C15-[Sur]-1	1058	25.62	212982 (90.12)	225572 (124.62)	100045 (65.57)	238515 (125.37)	430871 (78.18)	287165 (90.11)	370546 (77.56)	-
C14-[Val7]-4	1030	25.84	423288 (129.85)	122644 (146.62)	168556 (20.76)	264860 (153.48)	520643 (84.88)	199644 (95.72)	152503 (112.07)	220338 (59.81)
C15-[Sur]-2	1058	25.87	-	-	-	1541435 (140.12)	-	967869 (130.09)	1067664 (74.46)	-
C15-[AME5]-1	1072	25.89	51119 (40.00)	363436 (123.19)	68815 (88.89)	162336 (52.33)	76652 (17.40)	215281 (122.38)	-	-
C14-[Sur]-6	1044	26.23	-	-	-	-	-	-	-	7328458 (12.02)
C15-[Sur]-3	1058	26.25	2681879 (120.54)	1058351 (131.89)	1490390 (118.83)	-	3508858 (76.01)	91019 (120.71)	-	-
C14-[Val7]-5	1030	26.35	-	-	-	4935 (119.39)	-	-	-	-
C15-[AME5]-2	1072	26.39	770722 (83.44)	1181952 (135.07)	838870 (136.40)	944830 (100.15)	1443615 (75.00)	1281946 (76.71)	1718158 (73.31)	-
C15-[Sur]-4	1058	26.50	-	-	-	367408 (101.38)	352890 (81.80)	-	-	220128 (22.91)
C13-[Sur]-7	1030	26.60	-	-	-	-	-	-	-	725329 (31.93)
C14-[Sur]-8	1044	26.74	111370 (124.53)	34839 (114.30)	176136 (31.28)	99742 (105.37)	171177 (88.04)	-	-	1459297 (23.62)

C15-[Val7]-1	1044	26.74	-	81627 (102.63)	147816 (62.85)	-	101228 (79.99)	21129 (92.01)	41171 (28.96)	29173 (35.24)
C15-[AME5, Val7]-1	1058	26.75	-	306580 (141.13)	-	-	-	-	-	-
C14-[Val7]-7	1030	26.85	-	-	-	-	-	-	-	2681718 (5.30)
C15-[Val2,7]-1	1030	26.85	-	-	118350 (39.82)	56736 (136.25)	36404 (84.74)	-	-	-
C15-[Sur]-5	1058	26.88	-	-	720042 (24.61)	-	-	247433 (82.49)	353513 (31.17)	1231902 (119.72)
C15-[AME5]-3	1072	26.90	65792 (60.26)	123513 (127.22)	244469 (38.81)	139325 (65.40)	-	-	-	-
C15-[Val7]-2	1044	27.12	536381 (107.87)	254048 (108.31)	1212248 (32.73)	494356 (132.09)	807630 (82.91)	233648 (138.33)	459604 (64.47)	78593 (55.97)
C16-[Sur]-1	1072	27.15	-	-	767084 (19.19)	30188 (2.86)	-	544891 (16.98)	576198 (14.80)	-
C14-[Sur]-9	1044	27.24	-	-	343092 (42.01)	-	-	-	-	-
C15-[AME5]-4	1072	27.27	45902 (60.51)	74487 (81.15)	161295 (82.20)	-	81948 (78.41)	-	101379 (57.61)	-
C16-[Sur]-2	1072	27.40	269182 (109.17)	706409 (63.84)	-	362653 (81.60)	465726 (67.51)	-	-	-
C15-[Sur]-6	1058	27.51	-	-	67023 (91.70)	-	97373 (11.02)	15684 (89.06)	16193 (11.06)	4865387 (24.85)
C15-[AME5]-5	1072	27.52	-	87308 (41.70)	33465 (61.67)	54200 (106.56)	9943 (68.93)	13727 (73.23)	9495 (104.84)	390558 (71.67)
C16-[AME5]-1	1086	27.54	199194 (61.80)	1700188 (24.07)	1312892 (29.98)	1007206 (98.89)	458790 (98.93)	960349 (34.28)	665793 (36.63)	-
C14-[Val7]-8	1030	27.60	-	-	-	-	-	-	-	265106 (10.19)
C16-[Val7]-1	1058	27.89	-	-	143331 (41.76)	-	-	39450 (43.41)	54785 (46.94)	-
C16-[Sur]-3	1072	27.90	-	496539 (7.68)	636959 (7.73)	331330 (72.23)	625997 (19.65)	241358 (25.67)	237360 (14.62)	-
C15-[AME5]-6	1072	27.90	-	40520 (75.84)	37220 (33.18)	26978 (83.71)	66978 (86.94)	15556 (54.73)	21369 (71.44)	757553 (24.11)
C16-[Leu4, AME5]-1	1100	27.93	-	-	-	-	-	4143 (13.28)	-	-
C15-[Val7]-3	1044	28.00	-	-	-	-	15794 (51.77)	-	-	195635 (4.72)
C16-[Val2]-1	1058	28.01	-	179097 (2.40)	-	-	-	-	-	-
C14-[Val7]-9	1030	28.11	-	-	-	-	-	-	-	219511 (34.62)
C15-[Sur]-7	1058	28.14	-	-	-	-	-	-	16196 (28.00)	3053789 (22.45)
C16-[AME5]-2	1086	28.17	36518 (6.22)	311348 (17.93)	171578 (15.36)	194818 (101.28)	187653 (46.80)	90978 (70.97)	71279 (45.53)	-
C15-[Val7]-4	1044	28.25	16094 (80.06)	3642 (57.27)	-	-	10240 (5.06)	-	-	271012 (41.39)

C16-[Sur]-4	1072	28.40	351678 (52.50)	303712 (58.90)	607816 (57.16)	189533 (109.54)	467232 (37.51)	-	145815 (75.88)	75841 (109.29)
C14-[Sur]-10	1044	28.50	-	-	-	-	-	-	-	2086645 (8.94)
C15-[Sur]-8	1058	28.51	236241 (58.15)	141343 (73.57)	336629 (57.87)	121330 (117.29)	324727 (58.21)	41080 (145.65)	93396 (76.77)	714146 (3.20)
C16-[AME5]-3	1086	28.54	56227 (28.42)	202028 (43.84)	141196 (24.99)	102462 (72.12)	160588 (42.44)	48670 (11.65)	76741 (46.29)	-
C16-[Leu4, AME5]-2	1100	28.68	-	17670 (104.43)	6579 (66.74)	-	-	4869 (120.51)	-	-
C15-[Val7]-5	1044	28.87	-	-	-	-	-	-	-	2571130 (48.86)
C15-[AME5]-7	1072	28.91	-	-	-	-	-	-	-	197147 (83.38)
C17-[Sur]-1	1086	29.05	7181 (89.17)	44347 (17.44)	14254 (102.98)	29474 (75.40)	42995 (55.47)	8533 (33.75)	27946 (71.57)	-
C16-[Val7]-2	1058	29.14	24446 (25.06)	-	74214 (54.35)	-	-	-	22968 (111.96)	-
C16-[AME5]-4	1086	29.17	15989 (64.70)	110451 (19.10)	46002 (71.24)	65674 (140.08)	42086 (144.08)	55387 (71.32)	19072 (38.97)	65882 (37.78)
C14-[Sur]-11	1044	29.25	-	-	-	-	-	-	-	1216260 (31.39)
C16-[Sur]-6	1072	29.28	199153 (110.75)	31994 (60.30)	20848 (31.35)	14155 (81.31)	20711 (42.01)	15172 (115.09)	20860 (72.37)	-
C17-[AME5]-1	1100	29.44	15363 (14.83)	135506 (32.58)	81649 (42.68)	86845 (152.49)	49861 (129.96)	57374 (55.76)	32143 (90.82)	-
C15-[Val7]-6	1044	29.50	-	-	-	-	-	-	-	739412 (70.29)
C16-[Val7]-3	1058	29.52	18051 (108.81)	-	64367 (28.88)	-	-	-	9986 (126.57)	-
C16-[AME5]-5	1086	29.55	-	-	-	-	-	-	-	69226 (30.98)
C17-[Sur]-2	1086	29.67	15172 (60.12)	72264 (21.63)	52742 (67.96)	32914 (135.96)	41664 (30.01)	22395 (33.97)	29319 (24.65)	-
C16-[AME5]-6	1086	29.80	-	-	-	-	-	-	-	63971 (53.77)
C17-[AME5]-2	1100	29.81	19924 (33.96)	192066 (19.80)	59692 (72.98)	128765 (134.15)	58021 (126.22)	56834 (76.20)	26840 (19.06)	-
C17-[Sur]-3	1086	30.05	-	31759 (55.52)	12383 (108.90)	17741 (92.82)	957 (54.94)	9554 (58.35)	-	-
C17-[AME5]-3	1100	30.07	-	-	-	-	-	-	21300 (132.72)	-
C15-[Val7]-7	1044	30.13	-	-	-	-	-	-	-	304119 (18.70)
C16-[AME5]-7	1086	30.18	-	-	-	-	-	-	-	41107 (30.71)
C16-[Sur]-9	1072	30.29	5335 (50.34)	4373 (112.19)	12717 (43.83)	-	-	-	-	-
C15-[Val7]-8	1044	30.38	-	-	-	-	-	-	-	153666 (77.31)

C15-[Sur]-12	1058	30.40	-	-	-	-	-	-	-	4579835 (94.28)
C16-[AME5]-8	1086	30.43	-	-	-	-	-	-	-	23970 (9.16)
C17-[Sur]-4	1086	30.55	-	-	-	-	-	-	-	13825 (110.18)
C15-[Val7]-9	1044	30.63	-	-	-	-	-	-	-	193096 (98.56)
C17-[Sur]-5	1086	30.80	-	-	-	-	1354 (74.39)	-	-	29756 (134.08)
C17-[AME5]-4	1100	30.82	-	-	-	-	-	-	-	8162 (58.42)
C16-[AME5]-9	1086	30.93	-	-	-	-	-	-	-	23852 (66.95)
C17-[Sur]-6	1086	31.06	-	-	-	-	-	-	-	17643 (13.74)
C18-[AME5]-1	1114	31.09	-	4464 (149.87)	-	-	-	-	-	-
C16-[AME5]-10	1086	31.31	-	-	-	-	-	-	-	27205 (115.06)
C17-[Sur]-7	1086	31.43	-	-	-	-	-	-	-	158335 (123.95)
C17-[AME5]-5	1100	31.57	-	-	-	-	-	-	-	11370 (111.38)
C16-[AME5]-11	1086	31.68	-	-	-	-	-	-	-	11617 (100.90)
C17-[Sur]-8	1086	31.94	-	-	-	-	-	-	-	146983 (133.97)
C17-[AME5]-6	1100	31.95	-	-	-	-	-	-	-	3761 (87.39)
C16-[AME5]-12	1086	32.06	-	-	-	-	-	-	-	2611 (80.13)
C17-[AME5]-7	1100	32.20	-	-	-	-	-	-	-	3456 (125.86)
C17-[Sur]-9	1086	32.31	-	-	-	-	-	-	-	11695 (124.39)
C16-[AME5]-13	1086	32.56	-	-	-	-	-	-	-	2634 (125.02)
C16-[AME5]-14	1086	32.94	-	-	-	-	-	-	-	3830 (117.08)
C17-[Sur]-10	1086	33.07	-	-	-	-	-	-	-	6955 (111.79)
C16-[AME5, Val7]-1	1072	28.65	-	-	-	-	-	-	-	79880 (73.72)
C16-[Sur]-5	1072	28.90	-	-	-	-	-	-	-	337842 (15.38)
C15-[Sur]-9	1058	29.14	-	-	-	-	-	-	-	1304625 (43.79)
C16-[Sur]-7	1072	29.41	-	-	-	-	-	-	-	1123701 (84.37)

C15-[AME5]-8	1072	29.41	-	-	-	-	-	-	-	258011 (14.86)
C16-[Sur]-8	1072	29.78	-	-	-	-	-	-	-	395200 (29.25)
C15-[AME5]-9	1072	29.79	-	-	-	-	-	-	-	60727 (49.24)
C17-[Val2,7]-1	1058	29.90	-	-	-	-	-	-	-	4522 (43.21)
C17-[Val2,7]-2	1058	30.27	-	-	-	-	-	-	-	2061 (56.88)
C16-[Sur]-9	1072	30.29	-	-	-	-	-	-	-	391533 (35.23)
C15-[AME5]-10	1072	30.29	-	-	-	-	-	-	-	58979 (52.42)
C15-[AME5]-11	1072	30.54	-	-	-	-	-	-	-	56542 (136.34)
C16-[Sur]-11	1072	30.91	-	-	-	-	-	-	-	311103 (79.74)
C16-[Sur]-12	1072	31.29	-	-	-	-	-	-	-	160009 (130.24)
C15-[Sur]-14	1058	31.40	-	-	-	-	-	-	-	764079 (108.15)
C16-[Sur]-13	1072	31.92	-	-	-	-	-	-	-	141616 (93.08)
C16-[Sur]-14	1072	32.30	-	-	-	-	-	-	-	244600 (119.00)
C16-[Sur]-15	1072	32.80	-	-	-	-	-	-	-	340990 (130.48)

* RSD% are in brackets.
